# *Moringa oleifera* aqueous leaf extract inhibits reducing monosaccharide-induced protein glycation and oxidation of bovine serum albumin

**DOI:** 10.1186/s40064-016-2759-3

**Published:** 2016-07-16

**Authors:** Pornpimon Nunthanawanich, Weerachat Sompong, Sukrit Sirikwanpong, Kittana Mäkynen, Sirichai Adisakwattana, Winai Dahlan, Sathaporn Ngamukote

**Affiliations:** Graduate Program in Food and Nutrition, Department of Nutrition and Dietetics, Faculty of Allied Health Sciences, Chulalongkorn University, Bangkok, 10330 Thailand; Department of Nutrition and Dietetics, Faculty of Allied Health Sciences, Chulalongkorn University, Bangkok, 10330 Thailand; Research Group of Herbal Medicine for Prevention and Therapeutic of Metabolic Diseases, Chulalongkorn University, Bangkok, 10330 Thailand; The Halal Science Center, Chulalongkorn University, Bangkok, 10330 Thailand

**Keywords:** *Moringa oleifera*, Polyphenol, Glycation, Glucose, Fructose

## Abstract

Advanced glycation end products (AGEs) play an important factor for pathophysiology of diabetes and its complications. *Moringa oleifera* is one of the medicinal plants that have anti-hyperglycemic activity. However, anti-glycation property of *Moringa oleifera* leaf extract on the different types of reducing monosaccharides-induced protein glycation has not been investigated. Therefore, the aim of this study was to examine the protective effect of *Moringa oleifera* aqueous leaf extract (MOE) on reducing sugars-induced protein glycation and protein oxidation. Total phenolic content of MOE was measured using the Folin–Ciocalteu method. Bovine serum albumin was incubated with 0.5 M of reducing sugars (glucose or fructose) with or without MOE (0.5–2.0 mg/mL) for 1, 2, 3 and 4 weeks. The results found that total phenolic content was 38.56 ± 1.50 mg gallic acid equivalents/g dry extract. The formation of fluorescent and non-fluorescent AGEs [*N*^ε^-(carboxymethyl) lysine (CML)] and the level of fructosamine were determined to indicate protein glycation, whereas the level of protein carbonyl content and thiol group were examined for protein oxidation. MOE (0.5–2.0 mg/mL) significantly inhibited the formation of fluorescent, *N*^ε^-CML and markedly decreased fructosamine level (*P* < 0.05). Moreover, MOE significantly prevented protein oxidation manifested by reducing protein carbonyl and the depletion of protein thiol in a dose-dependent manner (*P* < 0.05). Thus, the findings indicated that polyphenols containing in MOE have high potential for decreasing protein glycation and protein oxidation that may delay or prevent AGE-related diabetic complications.

## Background

Advanced glycation end products (AGEs) are a complex of heterogeneous group of molecules that are formed from non-enzymatic glycation of carbonyl group of a reducing sugar with an amino group of proteins, lipids, or nucleic acids (Kaneko et al. 2005). The accumulation of AGEs in various types of tissues causes the alteration of proteins leading to change their characteristics, physiochemical, and biochemical properties (Vanessa et al. 2013). The interaction of AGEs with the receptor of advanced glycation end products evokes oxidative stress and subsequently elicits vascular inflammation and thrombosis (Kang 2003). Studies have also shown that reactive oxygen species (ROS) formed by AGEs cause DNA damage and induction of cell apoptosis (Kang 2003). Glucose and fructose, the most common reducing sugar found in blood circulation react spontaneously with amino groups of proteins to AGEs. Although glucose plays a vital role in the formation of AGEs, it is now known that fructose undergoes protein glycation much faster than glucose (Semchyshyn et al. 2014). Endogenous fructose production by the sorbitol pathway is also considered to contribute the formation of AGEs and consequently accumulate in the tissues (Suarez et al. 1989; Vinson and Howard 1996). There are several clinical studies which demonstrate the link between the long-term consumption of fructose and the development of aging process (Levi and Werman 1998). Therefore, there has been seriously concern regarding the critical role of fructose in the glycation process. Studies on antiglycating agents have recently emerged as the new therapeutic approaches in preventing AGE-related diseases (Adisakwattana et al. 2010). Aminoguanidine (AG) is one of therapeutic agents for use in the prevention of AGE formation by cleavage of AGE-induced chemical cross-links (Brownlee et al. 1986). However, it has shown serious side effects including vascularitis, gastrointestinal disturbances, and anemia (Brownlee et al. 1986). For this reason, the search for alternative prevention of AGE formation has been focused on the natural products.

Polyphenolic compounds are commonly found in vegetables, fruits, spices, and medicinal herbs. Previously, it has been shown that polyphenols play an important role in human health, including reduced risk of chronic and degenerative diseases (Vauzour et al. 2010; Ngamukote et al. 2011; Adisakwattana and Chanathong 2011). *Moringa oleifera* (Ma-rum) is the most widely cultivated species of a monogeneric family, the Moringaceae which is commonly found in tropical countries such as India, Afghanistan, as well as Thailand. In addition, Many studies have reported the flavonoid contents such as keamferol, quercetin, ferulic acid, gallic acid, rutin, caffeic acid as well as other phenolic compounds in multi-part of Moringa tree (Fahey 2005; Anwar et al. 2007). A number of previous studies have reported pharmacological properties of *Moringa oleifera* particular in antioxidant property and antidiabetic activity that may provide benefits for diabetic patients (Jaiswal et al. 2009; Chumark et al. 2008; Adisakwattana and Chanathong 2011). However, there are no reports in the literature showing that MOE can inhibit protein glycation induced by different types of reducing monosaccharaides in vitro models. Thus, the aim of present study was to evaluate the inhibitory effects of MOE on glucose- and fructose-induced protein glycation, about which no previous reports exist. Furthermore, the inhibitory effects of MOE on oxidation-dependent damages to bovine serum albumin mediated by glycation were also determined.

## Methods

### Chemicals

Glucose, fructose, and 2,4-dinitrophenyl hydrazine (DNPH) were purchased from Ajax Finechem (Taren Point, Australia). Catechin, gallic acid, sodium azide, Nitroblue tetrazolium (NBT), aminoguanidine hydrochloride (AG), guanidine hydrochloride, Thioflavin T (ThT), 5,5′-dithiobis(2-nitrobenzoic acid) (DTNB), and l-cysteine were obtained from Sigma-Aldrich Co. (St. Louis, MO, USA). Trichloroacetic acid (TCA) was purchased from Merck (Darmstadt, Germany). OxiSelectTM CML ELISA kit was purchased from Cell Biolabs (San Diego, CA, USA). All other reagents used were of analytical grade.

### Preparation of *Moringa oleifera* aqueous leaf extract

The leaves of *Moringa oleifera* were obtained from local areas of Bangkok in Nongkhame district, Thailand. The herbarium number of A014172 (BCU) was authenticated by a Taxonomist at Department of Botany, Faculty of Science, Chulalongkorn University, Thailand. The dried leaves (250 g) were extracted with distilled water twice (3 L) for 3 h at 100 °C. The extraction was filtered through Whatman No. 1 filter paper under the vacuum. The filtrate was further subjected to a spray dryer SD-100 (Eyela world, Tokyo Rikakikai Co., LTD, Japan) to obtain the extract powder. The spray drying conditions, inlet and outlet air temperature was set at 160 and 89–99 °C, respectively.

### Determination of total phenolic content

Total phenolic content of *Moringa oleifera* leaf extract (MOE) was determined by the Folin-Ciocalteu method (Verma et al. 2009). The extract powder was dissolved in distilled water (1.25 mg/mL). The freshly prepared Folin-Ciocalteu reagent was gently mixed with 10 µL of sample. Then, 75 µL of 7.5 % sodium carbonate (Na_2_CO_3_) was added and allowed to stand for 30 min at room temperature in the dark. The mixture was measured at 725 nm by a spectrophotometer. Gallic acid (0.025–0.4 mg/mL) was used as a standard and the content of total phenolics was expressed as mg gallic acid equivalents/g dried extract.

### Preparation of glycated bovine serum albumin (BSA)

Glycated BSA was performed according to a previously described method (Povichit et al. 2010) with slight modifications. Briefly, BSA (10 mg/mL) was incubated with glucose or fructose (0.5 M) in 0.1 M phosphate buffer (pH 7.4) containing 0.02 % sodium azide (NaN_3_) with or without (MOE) (0.5–2.0 mg/mL) and aminoguanidine (AG, 1.0 mg/mL) at 37 °C for 4 weeks. Samples were kept at −20 °C until analysis.

### Determination of advanced glycation end product (AGE) formation

The fluorescent AGEs, the irreversible products at the end stage of non-enzymatic glycation, were determined by a spectrofluorometer (Wallac 1420 Victor3 V, PerkinElmer, Santa Clara, CA, USA) at excitation and emission wavelengths of 355 nm and 460 nm, respectively (Povichit et al. 2010).

### Determination of *N*^ε^-(carboxymethyl) lysine (CML)

Non-fluorescent AGEs, *N*^ε^-(carboxymethyl) lysine (*N*^ε^-CML), is the most abundant product of glycation reaction. Commercially available ELISA kit was used for measurement of *N*^ε^-CMLformation (Cell Biolabs, CA, USA).

### Determination of fructosamine

The levels of fructosamine was analyzed by nitroblue-tetrazolium (NBT) assay with minor modification (Armbruster 1987). Briefly, 90 µL of 2.5 mM nitrobluetetrazolium (NBT) reagent was added to 10 µL of glycated BSA in carbonate buffer (pH 10.3). After 10 and 15 min of incubation, the mixture was measured at 590 nm. The concentration of fructosamine was calculated by using the different absorption at 10 and 15 min time points compared with the standard 1-deoxy-1-morpholino-fructose (1-DMF) curve.

### Determination of protein carbonyl

Protein carbonyl content were determined according to a previously published method with minor modifications (Levine et al. 1990). In brief, 10 mM 2,4-dinitrophenylhydrazine (DNPH) in 2.5 M HCl (400 µL) was added to 100 µL of glycated BSA and incubated in the dark at room temperature for 60 min. Then, 20 % (w/v) trichloroacetic acid (500 µL) was added and kept on ice for 5 min. Protein precipitation was centrifuged and the protein pellet was then washed with 1:1 (v/v) ethanol/ethyl acetate and dissolved in 6 M guanidine hydrochloride. The absorbance was determined at 370 nm. The concentration of protein carbonyl content was calculated using an absorption coefficient of 22,000 M^−1^cm^−1^. The results were expressed as nmol carbonyl/mg protein.

### Determination of protein thiol groups

The determination of free thiol groups were performed according to Ellman’s assay using 5,5′-dithiobis-(2-nitrobenzoic acid) (DTNB) (Ellman 1959). Glycated BSA (10 µL) was incubated with 6 mM DTNB in 0.1 M PBS (pH 7.4) for 15 min at room temperature. The absorbance was determined at 410 nm. The free thiol concentration was calculated for the standard curve of l-cysteine (0.3–10 µM) and expressed as nmol/mg protein.

### Statistical analysis

Data are expressed as mean ± standard error of mean (SEM) of triplicate determination (n = 3). Differences among groups were analyzed for statistical significance by one-way ANOVA followed by Duncan as post hoc comparison. *P* value < 0.05 was considered statistically significant.

## Results

### Phytochemical analysis

In the present study, the content of total phenolic compounds in *Moringa oleifera* leaf extract (MOE) was 38.56 ± 1.50 mg gallic acid equivalents/g extract.

### Effect of *Moringa oleifera* leaf extract on the different types of reducing monosaccharide-induced fluorescent AGE formation

**Fig. 1:**
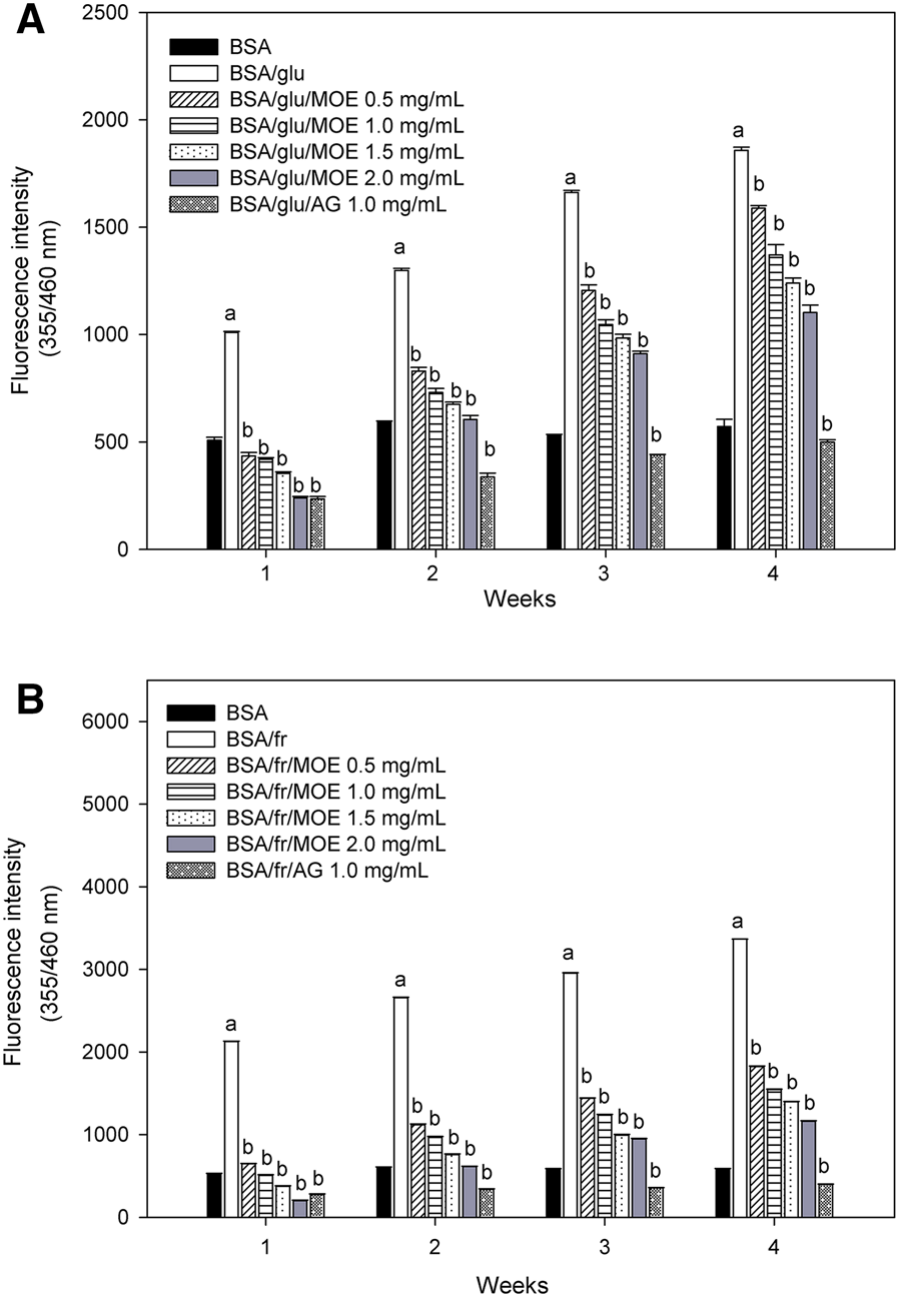
The effect of *Moringa oleifera* leaf extract (MOE, 0.5–2.0 mg/mL) and aminoguanidine (AG, 1.0 mg/mL) on the formation of fluorescent AGEs in **A** bovine serum albumin (BSA) incubated with 0.5 M glucose (glu) and **B** bovine serum albumin (BSA) incubated with 0.5 M fructose (fr) at week 1, 2, 3 and 4 of incubation. The results are expressed as mean ± SEM (n = 3). ^a^ *P* < 0.05 when compared to BSA alone, ^b^ *P* < 0.05 when compared to BSA with glucose (BSA/glu) or BSA with fructose (BSA/fr) at the same week of incubation

The formation of fluorescent AGEs in different monosaccharide-induced protein glycation was monitored during 4 weeks of incubation. As shown in Fig. 1, the significant increase in fluorescent intensity in BSA incubated with glucose and fructose was seen during 4 weeks of the incubation. The results demonstrated that the fluorescent AGE formation was increased 3.24-fold in glucose model (Fig. 1A) and 5.76-fold in fructose model (Fig. 1B) whereas MOE (0.5–2.0 mg/mL) inhibited the formation of AGEs in a dose-dependent manner during experimental periods both glucose and fructose models at week 4. The percentage inhibition of AGE formation by MOE (0.5–2.0 mg/mL) ranged from 14.52–40.65 % in glucose-glycated BSA and 45.82–65.43 % in fructose-glycated BSA. However, MOE has less potent in the inhibition of AGE formation when compared with AG at the same concentration (1 mg/mL).

### Effect of *Moringa oleifera* leaf extract on the level of *N*^ε^-(carboxymethyl) lysine (CML)

**Fig. 2:**
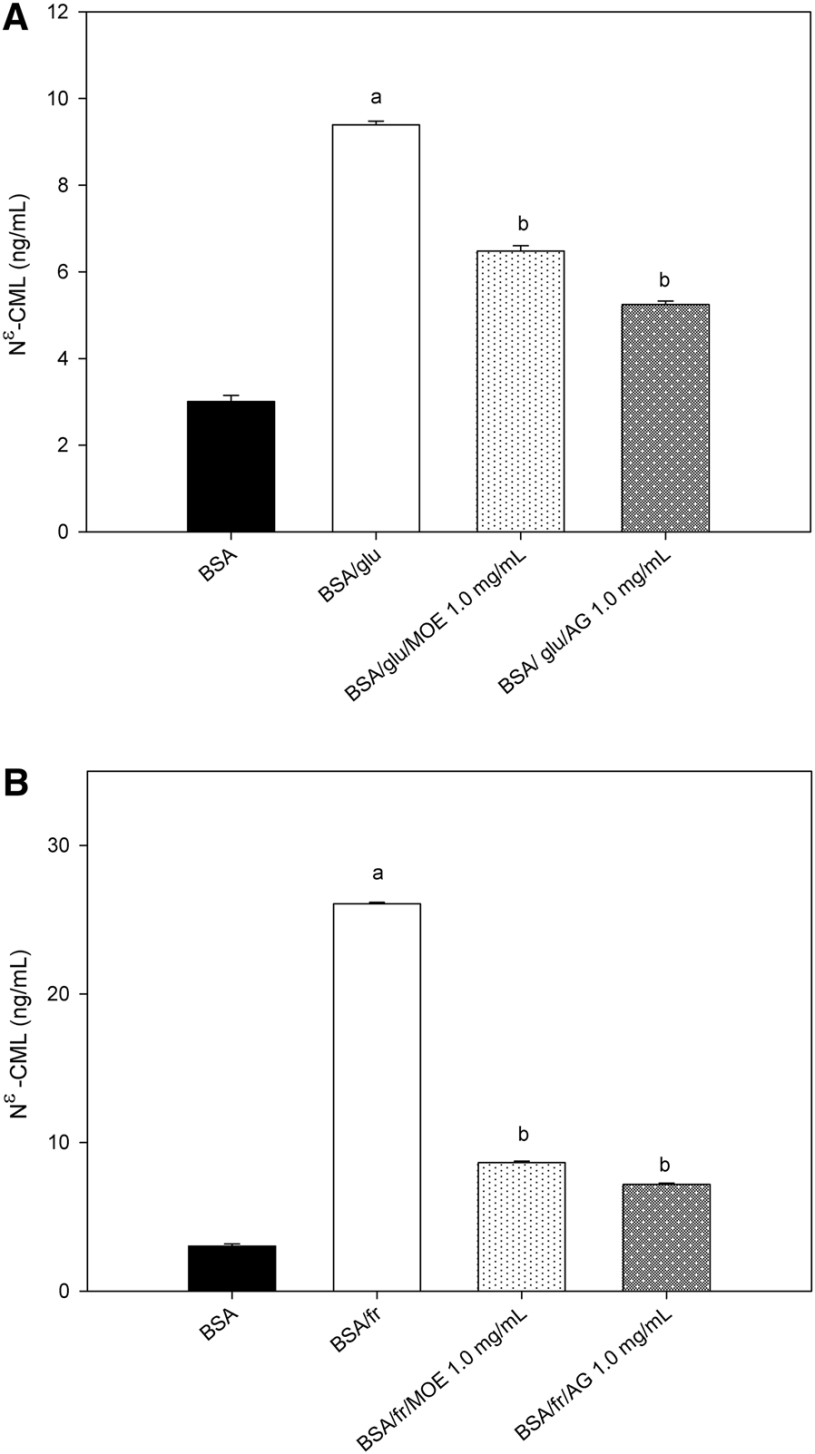
The effect of *Moringa oleifera* leaf extract (MOE, 1.0 mg/mL) and aminoguanidine (AG, 1.0 mg/mL) on the level of *N* ^ε^-(carboxymethyl) lysine (CML) in **A** bovine serum albumin (BSA) incubated with 0.5 M glucose (glu) and **B** bovine serum albumin (BSA) incubated with 0.5 M fructose (fr) at week 4 of incubation. The results are expressed as mean ± SEM (n = 3). ^a^ *P* < 0.05 when compared to BSA alone, ^b^ *P* < 0.05 when compared to BSA with glucose (BSA/glu) or BSA with fructose (BSA/fr) at the same week of incubation

In order to examine the formation of non-fluorescent AGEs, the level of *N*^ε^-CML was measured at week 4 of incubation. As shown in Fig. 2, it was found that the level of *N*^ε^-CML dramatically increased in glucose-glycated BSA (3.10-fold) and fructose-glycated BSA (8.60-fold). Conversely, the addition of MOE to the solution (1.0 mg/mL) inhibited *N*^ε^-CML formation about 31.02 % in glucose-glycated BSA and about 66.82 % in fructose-glycated BSA, whereas AG (1.0 mg/mL) inhibited *N*^ε^-CML formation about 44.17 % in glucose-glycated BSA and about 72.45 % in fructose-glycated BSA.

### Effect of *Moringa oleifera* leaf extract on the level of fructosamine

**Fig. 3:**
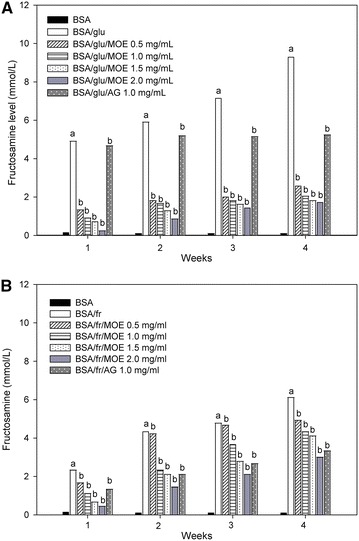
The effect of *Moringa oleifera* leaf extract (MOE, 0.5–2.0 mg/mL) and aminoguanidine (AG, 1.0 mg/mL) on fructosamine level in **A** bovine serum albumin (BSA) incubated with 0.5 M glucose (glu) and **B** bovine serum albumin (BSA) incubated with 0.5 M fructose (fr) at week 1, 2, 3 and 4 of incubation. The results are expressed as mean ± SEM (n = 3). ^a^ *P* < 0.05 when compared to BSA alone, ^b^ *P* < 0.05 when compared to BSA with glucose (BSA/glu) or BSA with fructose (BSA/fr) at the same week of incubation

The effects of MOE on the level of fructosamine are shown in Fig. 3. The level of fructosamine in glucose-glycated BSA and fructose-glycated BSA markedly increased throughout 4 weeks of the experiment. In contrast, the increasing level of fructosamine was attenuated by MOE (0.5–2.0 mg/mL) during 4 weeks of the study. At week 4 of incubation, MOE (0.5–2.0 mg/mL) reduced the level of fructosamine in a concentration-dependent manner in glucose-glycated BSA (71.21–80.30 %) and fructose-glycated BSA (18.96–49.56 %). In addition, the percentage reduction of fructosamine by AG (1 mg/mL) was 42.92 % for glucose-glycated BSA and 45.45 % for fructose-glycated BSA.

### Effect of *Moringa oleifera* leaf extract on the level of protein carbonyl content and protein thiol groups

**Fig. 4:**
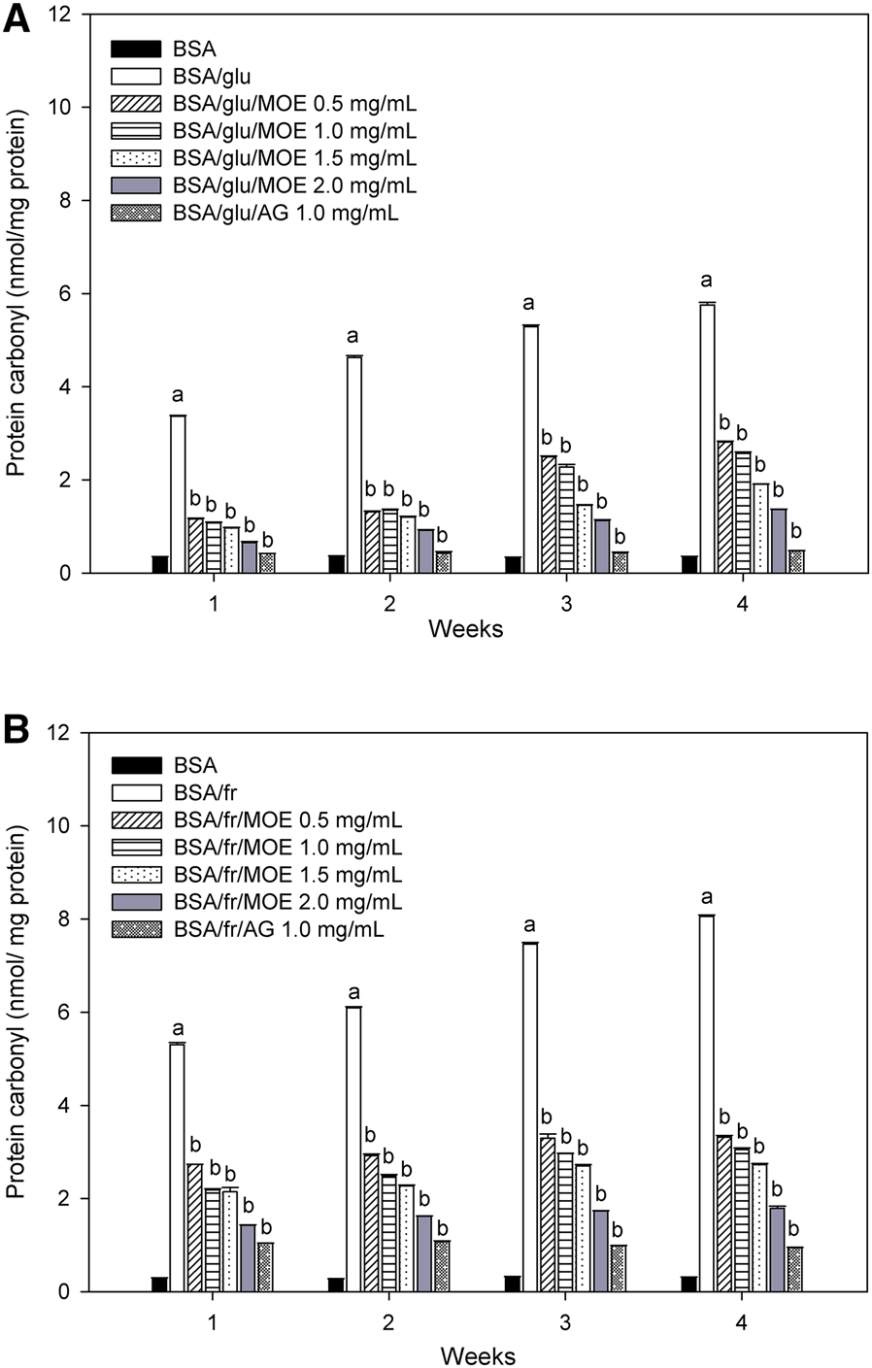
The effect of *Moringa oleifera* leaf extract (MOE, 0.5–2.0 mg/mL) and aminoguanidine (AG, 1.0 mg/mL) on protein carbonyl content in **A** bovine serum albumin (BSA) incubated with 0.5 M glucose (glu) and **B** bovine serum albumin (BSA) incubated with 0.5 M fructose (fr) at week 1, 2, 3 and 4 of incubation. The results are expressed as mean ± SEM (n = 3). ^a^ *P* < 0.05 compared to BSA alone, ^b^ *P* < 0.05 when compared to BSA with glucose (BSA/glu) or BSA with fructose (BSA/fr) at the same week of incubation

**Fig. 5:**
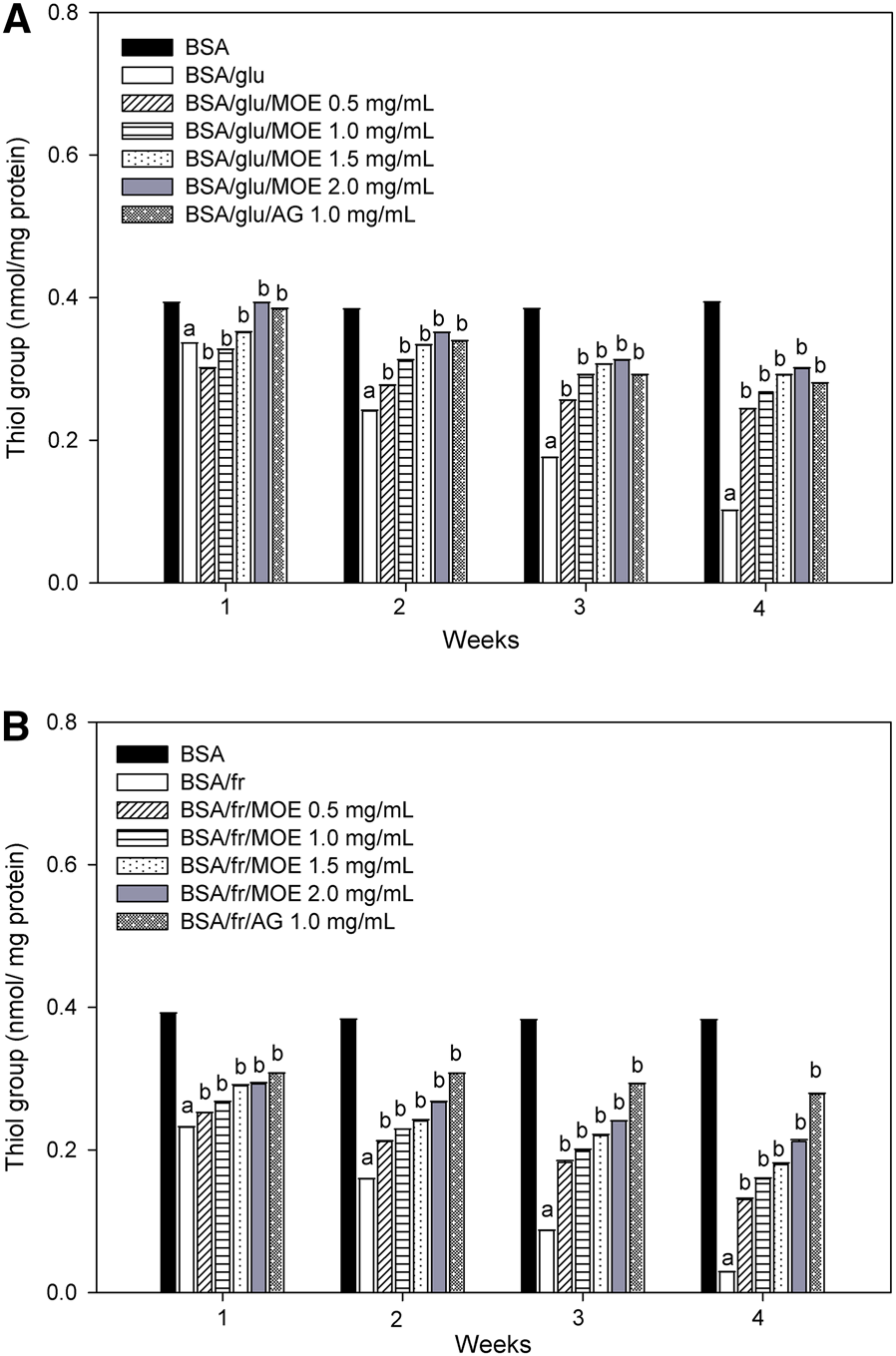
The effects of *Moringa oleifera* leaf extract (MOE, 0.5–2.0 mg/mL) and aminoguanidine (AG, 1.0 mg/mL) on thiol group in **A** bovine serum albumin (BSA) incubated with 0.5 M glucose (glu) and **B** bovine serum albumin (BSA) incubated with 0.5 M fructose (fr) at week 1, 2, 3 and 4 of incubation. The results are expressed as mean ± SEM (n = 3).  ^a^ *P* < 0.05 compared to BSA alone, ^b^ *P* < 0.05 when compared to BSA with glucose (BSA/glu) or BSA with fructose (BSA/fr) at the same week of incubation

The level of carbonyl content and thiol groups were used for indication of the protein oxidation mediated by glycation process. As shown in Fig. 4, the carbonyl content of glucose-glycated BSA and fructose-glycated BSA significantly increased during the experimental period, whereas MOE (0.5–2.0 mg/mL) significantly suppressed an increase in protein carbonyl content of glucose-glycated BSA and fructose-glycated BSA. When comparing with glucose-glycated BSA and fructose-glycated BSA at week 4, the percentage reduction of carbonyl content by MOE (0.5–2.0 mg/mL) ranged from 51.12 to 76.31 % in glucose-glycated BSA and 58.79 to 77.76 % in fructose-glycated BSA. A significant reduction of protein carbonyl content (91.59 % for glucose-glycated BSA and 88.29 % for fructose-glycated BSA) was observed in the presence of AG (1 mg/mL) at the same week. The effects of MOE on the oxidation of protein thiols are presented in Fig. 5. When BSA was incubated with glucose or fructose, the level of thiol groups had continuously declined throughout the experimental periods. Interestingly, there was a significant increase in the level of thiol groups after addition of MOE (0.5–2.0 mg/mL) as well as AG (1.0 mg/mL). The findings showed that the percentage prevention of depleting thiol group by MOE ranged from 7.57 to 9.77 % in glucose-glycated BSA and 5.73 to 10.32 % in fructose-glycated BSA, whereas AG (1.0 mg/mL) significantly prevented the depletion of protein thiol groups around 9.46 % and 14.09 % in glucose-glycated BSA and fructose-glycated BSA, respectively at the week 4.

## Discussion

In a present study, MOE was investigated the effect on glucose- and fructose-induced florescent and non-fluorescent AGE formation. The results showed that MOE efficiently inhibited fluorescent and non-fluorescent AGE formation. MOE also reduced the level of fructosamine associated with the reduction of AGE formation in glucose-glycated BSA and fructose-glycated BSA. A significant decrease of protein carbonyl content and oxidation of thiols in BSA were seen when MOE was added to the systems, it markedly suppressed these processes. The blockage of the carbonyl group in reducing sugars, the trapping of reactive oxygen species (ROS) and carbonyls during glycation, and breaking the crosslinking structure in the formed AGEs have recently been revealed as the underlying mechanisms of antiglycating agents (Price et al. 2001). The scavenging free radical generation during glycation process may highlight other mechanisms for the prevention of AGE formation (Rout and Banerjee 2007). The Amadori products can be fragmented and consequently generate superoxide anion to form fluorescent and non-fluorescent AGEs at the early stage of glycation (Peyroux and Sternberg 2006). An excess production of ROS causes oxidative damage to proteins which lead to introduce carbonyl groups on the side chain of proteins and deplete thiol group of protein (Dalle-Donne et al. 2003).

Recent studies have shown that polyphenolic compounds from the edible plants play a vital role to protect monosaccharide-induced protein glycation and oxidation (Adisakwattana et al. 2010). Additionally, there is a strong link between the polyphenolic content in the tested plant extracts and the ability to inhibit AGE formation (Peng et al. 2011; Wu and Yen 2005). Evidence also supports that the inhibitory effect of polyphenols against protein glycation is strongly related to their ability of scavenging free radical derived from the glycoxidation process (Povichit et al. 2010). Our findings indicate that MOE has high content of polyphenolic compounds. The content of total phenolic compounds in *Moringa oleifera* leaf extract (MOE) was consistent with previous studies that the content of total phenolic compounds in MOE ranged 33.82–45.21 mg gallic acid equivalents/g extract. According to the results obtained, we addressed the hypothesis that polyphenolic compounds in MOE may be a major contributor to inhibit the formation of AGEs. Previous studies have demonstrated that MOE has antioxidant activity against free radicals including DPPH free radical, superoxide, hydroxyl and nitric oxide radical (Siddhuraju and Becker 2003). Therefore, the inhibitory effect of MOE on glycation-induced protein oxidation may be due to its antioxidant properties. However, certain active biological constituents of MOE remain unknown. To prove this hypothesis, separation and characterization of polyphenolic compound in MOE using HPLC–MS are required for further study.

## Conclusion

*Moringa oleifera* aqueous leaf extract effectively inhibits reducing monosaccharide-induced AGE formation, protein oxidation and protein cross-linking in glycation reaction. This finding could be suggested that *Moringa oleifera* aqueous leaf extract may be used as an antiglycation agent to prevent the progression of diabetic complications.

## Abbreviations

AGEs: advanced glycation end products
*N*^ε^-CML: *N*^ε^-(carboxymethyl) lysine
BSA: bovine serum albumin
AG: aminoguanidine
MOE: *Moringa oleifera* leaf extract
